# The molecular spectrum and distribution of haemoglobinopathies in Cyprus: a 20-year retrospective study

**DOI:** 10.1038/srep26371

**Published:** 2016-05-20

**Authors:** Petros Kountouris, Ioanna Kousiappa, Thessalia Papasavva, George Christopoulos, Eleni Pavlou, Miranda Petrou, Xenia Feleki, Eleni Karitzie, Marios Phylactides, Pavlos Fanis, Carsten W. Lederer, Andreani R. Kyrri, Eleni Kalogerou, Christiana Makariou, Christiana Ioannou, Loukas Kythreotis, Georgia Hadjilambi, Nicoletta Andreou, Evangelia Pangalou, Irene Savvidou, Michael Angastiniotis, Michael Hadjigavriel, Maria Sitarou, Annita Kolnagou, Marina Kleanthous, Soteroula Christou

**Affiliations:** 1 Molecular Genetics Thalassaemia, The Cyprus Institute of Neurology and Genetics, 6 International Airport Ave., 2370 Nicosia, Cyprus; 2 Thalassemia Screening Laboratory, Thalassemia Center, Archbishop Makarios III Hospital, 1474 Nicosia, Cyprus; 3 Thalassemia Center, Archbishop Makarios III Hospital, 1474 Nicosia, Cyprus; 4 Thalassaemia International Federation, 31 Ifigeneias Street, 2007 Strovolos, Nicosia, Cyprus; 5 Thalassemia Center, Limassol General Hospital, 3304 Limassol, Cyprus; 6 Thalassemia Center, Larnaca General Hospital, 6301 Larnaca, Cyprus; 7 Thalassemia Center, Paphos General Hospital, 8100 Paphos, Cyprus

## Abstract

Haemoglobinopathies are the most common monogenic diseases, posing a major public health challenge worldwide. Cyprus has one the highest prevalences of thalassaemia in the world and has been the first country to introduce a successful population-wide prevention programme, based on premarital screening. In this study, we report the most significant and comprehensive update on the status of haemoglobinopathies in Cyprus for at least two decades. First, we identified and analysed all known 592 β-thalassaemia patients and 595 Hb H disease patients in Cyprus. Moreover, we report the molecular spectrum of α-, β- and δ-globin gene mutations in the population and their geographic distribution, using a set of 13824 carriers genotyped from 1995 to 2015, and estimate relative allele frequencies in carriers of β- and δ-globin gene mutations. Notably, several mutations are reported for the first time in the Cypriot population, whereas important differences are observed in the distribution of mutations across different districts of the island.

The haemoglobin (Hb) molecule is responsible for binding and transport of oxygen and carbon dioxide by red blood cells and is critical for their shape, integrity and half-life. The Hb protein complex consists of two α-like chains, encoded by genes in the α-locus on chromosome 16 (RefSeq ID: NG_000006), namely ζ (*HBZ*), α1 (*HBA1*) and α2 (*HBA2*), and two β-like chains, encoded by genes in the β-locus on chromosome 11 (RefSeq ID: NG_000007), namely ε (*HBE*), Aγ (*HBG1*), Gγ (*HBG2*), δ (*HBD*) and β (*HBB*). Haemoglobinopathies are caused by mutations in the two globin gene clusters and are characterised by a reduced or absent synthesis of globin chains in the case of the thalassaemia syndromes, mainly α- and β-thalassaemia, or by defects in the Hb protein structure in the case of structural Hb variants, such as the Hb S that causes sickle-cell disease. To date, more than 1865 disease-causing mutations have been reported, affecting different levels of gene regulation and expression^1^.

Haemoglobinopathies are the most common monogenic diseases in the world^2^ and are prevalent in former malaria regions in the Mediterranean, the Middle-East, South-East Africa and Sub-Saharan Africa^3^. Cyprus is an Eastern Mediterranean island where an exceptionally high incidence of thalassaemia poses a major public health challenge. More specifically, the prevalence of β-thalassaemia patients was estimated to be around 1 in 1000^4^, while the β-thalassaemia carrier rate was recently estimated to be around 12%^5^, with earlier estimates in the range of 15–18%^6^, one of the highest in the world. In addition, the α-thalassaemia carrier rate was estimated to be around 20%^7^, although earlier studies using electrophoresis estimated it to be about 10–12% of the population^8^,^9^. Cyprus was the first country to introduce a successful population-wide prevention programme for β-thalassaemia, based on premarital screening, and, as a result, the annual birth rate has decreased to less than five cases from an expected 30–50^6^,^10^.

α-thalassaemia is characterised by a decrease or complete absence of expression from one or more of the four α-globin genes and may be brought about by a deletion or a nondeletion mutation in the α-globin genes. Mutations are divided into two major classes: (a) α^0^-thalassaemia mutations, which delete both α-globin genes on the same chromosome, and (b) α^+^-thalassaemia mutations, which delete or deactivate only one of the α-globin genes. As a result, four clinical conditions of increased severity are recognised, based on the number of α-globin genes affected^11^,^12^: (a) α-thalassaemia silent carrier, in which only one α-globin gene is affected by a deletion (-α/αα) or a nondeletion (α^ND^α/αα) and which is mainly asymptomatic, (b) α-thalassaemia trait, in which only two α-globin genes are functional, either in *cis* (--/αα) or in *trans* (-α/-α or α^ND^α/α^ND^α), and which usually results in mild anaemia, (c) Hb H disease, in which there is only one functional α-globin gene (--/-α or --/α^ND^α), and (d) Hb Bart’s hydrops fetalis, in which all α-globin genes are deleted (--/--), leading to severe anaemia and, usually, death *in utero* or shortly after birth. The spectrum of α-thalassaemia mutations has been well-documented over the last decades^13^, with more than 230 mutations currently reported in the public IthaGenes database^1^. In Cyprus, the α-thalassaemia carrier rate and the relative allele frequencies were previously determined by screening 495 random cord blood samples^7^. The -α3.7 deletion is the most common α-globin mutation, accounting for 72.8% of all α-globin mutations, while the most common α^0^ mutations, specifically -α20.5 and --MED I, account for a combined 7.8% of all α-globin mutations and about 0.8% of the population. Consequently and despite the high prevalence of α-thalassaemia carriers in Cyprus, the risk for Hb Bart’s hydrops fetalis is relatively low, due to the low prevalence of α^0^ mutations in the population. In contrast, the number of Hb H disease patients is higher, but no study has ever determined the Hb H disease prevalence in the population, an omission that is all the more striking given that a wide range of severe phenotypic characteristics have been observed, particularly in the nondeletional type of the disease^14^,^15^.

β-thalassaemia is characterised by the reduced synthesis (β^+^) or absence (β^o^) of the β-globin chains in the Hb molecule, resulting in accumulation of unbound α-globin chains that precipitate in erythroid precursors in the bone marrow and in the mature erythrocytes, leading to ineffective erythropoiesis and peripheral haemolysis^16^. It is mainly caused by single nucleotide substitutions, small deletions or insertions within the β-globin gene or its immediate flanking sequence and, rarely, by large deletions^17^,^18^. To date, more than 350 β-thalassaemia mutations have been reported in the IthaGenes database^1^. In Cyprus, the most common β*-*thalassaemia mutation is IVS I-110 (G>A), with a frequency of 74–80%, followed by three other alleles, specifically IVS II-745 (C>G), IVS I-6 (T>C), IVS I-1 (G>A), with frequencies of 5–8%^19^.

In adult life, Hb A (α_2_β_2_) is the major Hb component with Hb A_2_ (α_2_δ_2_) represented in a fraction of 2.5–3.5% and with traces (<1%) of Hb F (α_2_γ_2_). The percentage of Hb A_2_ is usually higher in β-thalassaemia carriers, because of the reduced production of Hb A, while it is usually lower for Hb H disease patients. Therefore and although δ-thalassaemia has no clinical significance, mutations in the δ-globin gene interfere with typical thalassaemia phenotypic characteristics, affecting population screening for thalassaemia. Thus, investigation of δ-globin gene mutations in the Cypriot population is important to avoid misdiagnosis for thalassaemia carriers^20^. In a previous study^21^, the carrier frequency for mutant δ-globin chromosomes was estimated to be around 1.26% and the spectrum of observed δ-globin gene mutations was reported for the Cypriot population.

In addition to the thalassaemias, several less prevalent structural Hb variants have been identified in the Cypriot population^22^. Nine structural variants concerning the β-globin chains and three concerning the α-globin chains have been identified, with the most common being Hb S (0.2%), Hb D-Punjab (0.02%), Hb Lepore Boston-Washington (0.03%) for the β-globin chain and Hb Setif (0.1%) for the α-globin chain. Recently, a novel δ-globin chain variant (Hb A_2_-Famagusta) was discovered in four distinct families in Cyprus^23^, while other δ-globin variants have been observed in the past^21^, with Hb A_2_-Yialousa being the most prevalent.

This article reports (a) the molecular spectrum and geographic distribution of all known β-thalassaemia patients and Hb H disease patients in Cyprus, (b) the molecular spectrum of α-, β- and δ-globin gene mutations in the population and their geographic distribution, and (c) an updated, more precise estimation of the relative allele frequencies in carriers of β- and δ-globin gene mutations. In this retrospective investigation, we retrieved and analysed genotypic characteristics of samples isolated during the last 20 years from 592 β-thalassaemia patients, 595 Hb H disease patients and 13824 carriers of α-, β- and δ*-*globin gene mutations. Therefore, this study represents the most significant and comprehensive update on the status of haemoglobinopathies in Cyprus for at least two decades, providing comprehensive evidence for the success and critical information for the improvement of the population screening programme.

## Materials and Methods

### Ethics Statement

The study is in accordance with the guidelines and regulations of the Cyprus legislation and National Bioethics Committee. All genetic and personal information used throughout this study were collected as part of the routine diagnostic services at the Cyprus Institute of Neurology and Genetics (CING) from 1995 to 2015, after the request of the participants and in accordance with the CING regulations, whereas no additional data were collected or stored for this research investigation. All subjects were de-identified in compliance with the FDA Guidance Document “Informed Consent for *In Vitro* Diagnostic Device Studies Using Leftover Human Specimens that are Not Individually Identifiable” issued in April 2006 and is exempt from IRB review, as also confirmed by the Cyprus National Bioethics Committee. In addition, the study is in accordance with the guidelines provided in 2002 by the Council for International Organizations of Medical Sciences (CIOMS) in collaboration with the World Health Organization (WHO), as well as with the Working Party document 131 “Working Document on the processing of personal data relating to health in electronic health records (EHR)” (00323/07/EN WP 131), published in 2007 (under Article 29 of Directive 95/46/EC). To preserve the anonymity of these subjects, demographic data were limited to geographic distribution at the low-resolution district-level, age and sex, while only summary data are reported without providing detailed descriptions of the individual cases.

### Study design and subjects

Since 1978, population screening for haemoglobinopathies in Cyprus has been performed by the Thalassaemia Screening Laboratory of the Cyprus Thalassaemia Centre at Nicosia. Selected samples with abnormal haematological indices have been referred to the Molecular Genetics Thalassaemia department in the CING for molecular characterisation and identification of haemoglobinopathy carriers, whereas additional individuals have been referred for molecular analysis as part of family studies. Moreover, genetic analysis was performed as part of prenatal diagnosis for couples at risk of an affected thalassaemia birth. In addition and through its role as the reference centre for genetic testing for haemoglobinopathies, the CING performed genetic analysis for all known β-thalassaemia patients and Hb H disease patients in Cyprus.

This study includes data for Greek Cypriots, who, according to the latest population census in 2011 (http://www.mof.gov.cy/mof/cystat/statistics.nsf/census-2011_cystat_en/census-2011_cystat_en), represent about 98.8% of the habitants with Cypriot citizenship. Owing to the political situation, only sporadic samples of Turkish Cypriots were analysed as part of the national control programme of the Republic of Cyprus, even though they would normally account for a significant fraction of the population. To allow a clear definition of the sample population and of the scope of this study, those sporadic samples were thus not included in our analyses.

This study includes data for all β-thalassaemia patients and Hb H patients managed by the dedicated Thalassaemia Clinics in all four cities, specifically Nicosia, Limassol, Larnaca (merged with the smaller Famagusta district) and Paphos, as well as carrier data obtained by the CING from 1995 to 2015, as part of the routine genetic analysis for haemoglobinopathies. After removing transplanted and deceased patients, we compiled two datasets of thalassaemia patients, specifically (a) 592 β-thalassaemia patients, and (b) 595 Hb H disease patients. In addition, we compiled a dataset of all carriers genotyped at the CING from 1995 to 2015, resulting in 13824 individuals, more specifically overlapping sets of (a) 9287 carriers of α-globin gene mutations, namely individuals with silent α-thalassaemia and α-thalassaemia trait, (b) 4700 carriers of a β-globin gene mutation, and (c) 504 individuals with one or both δ-globin genes mutated. To avoid reduncancies in the datasets, we selected only unrelated individuals for the analysis of α- and δ-globin genes, resulting in final datasets of 8412 carriers of α-globin gene mutations and 428 carriers of δ-globin gene mutations. In the case of β-thalassaemia carriers, we compiled a random dataset from couples at risk of having an affected birth that participated in prenatal diagnosis, giving a final dataset of 2335 unrelated β-thalassaemia carriers. Sample sizes, age and sex distribution for all datasets are summarised in Table 1.

### DNA isolation and genotyping of α-, β- and δ-globin genes

Genomic DNA was extracted from peripheral blood using the Gentra Puregene Kit (Qiagen, Valencia, CA, USA) according to the manufacturer’s protocol and kept at −80 °C for long-term storage. For genotyping of the causative pathological β*-*globin mutations, we used the single-tube amplification refractory mutation system-Polymerase Chain Reaction (ARMS-PCR) methodology^24^. Seven mutations common to the Cypriot population were tested, specifically IVS I-110 (G>A), IVS I-6 (T>C), IVS I-1 (G>A), IVS II-745 (C>G), CD 39 (C>T), -87 (C>G) and CD 5 (-CT). When no mutation was detected by the ARMS-PCR, the β-globin gene was examined by performing PCR and Sanger sequencing using an ABI 3130xl Genetic Analyzer (Applied Biosystems-Life Technologies, USA). Deletion-type mutations were tested using the multiplex ligation-dependent probe amplification method (MLPA) by utilising the SALSA MLPA probemix P102 HBB protocol (MRC, Holland).

The samples of β-thalassaemia patients, Hb H disease patients and carriers of α-globin gene mutations were investigated for α-globin deletions and/or point mutations by gap-PCR genotyping assays. The α-thalassaemia screening panel consisted of deletions and point mutations representing the common α-thalassaemia determinants encountered previously in Cyprus, specifically -α3.7, triplicated α (ααα or anti^−α3.7^)^25^, --MED I, -α20.5, IVS I-1 (-5 bp), Poly(A) AATAAA >AATGAA and Hb Agrinio^15^,^26^. In the case of Hb H disease patients and when no mutation was detected by the gap-PCRs, the α-globin genes were studied by sequencing, as detailed above for β-globin gene mutations. Deletion-type mutations were tested using the MLPA method by utilising the SALSA MLPA probemix P140-B3 HBA protocol (MRC, Holland). Protocols for these procedures are described in detail in the IthaPedia wiki at the ITHANET Portal (www.ithanet.eu/ithapedia)^27^. In addition, possible carriers of δ-globin gene mutations were tested by performing PCR and Sanger sequencing as described elsewhere^21^.

### Statistical analysis

The data were analysed using the *R* programming language (version 3.2.4). The analysis included data manipulation, filtering and plotting using the R packages *tidyr, dplyr* and *ggplot2*. Descriptive statistics were utilised for the analysis, particularly to calculate mutation frequencies for haemoglobinopathies in Cyprus and in individual districts. In addition, the one-sample proportions test (Wilson score) with Yates’ continuity correction was utilised to calculate 95% confidence intervals (95% CI), using the *prop.test* function in R.

## Results and Discussion

### Carriers of α-globin gene mutations

From 1995 to 2015, 9287 carriers of α-globin gene mutations were genotyped at the CING. After removing related individuals with identical genotype, the remaining 8412 carriers were used to analyse the molecular spectrum and distribution of α-globin gene mutations, shown in Table 2. However, we do not report the relative allele frequencies, because the dataset of α-globin gene mutation carriers referred to the CING for genotyping is not a random representation of the carrier population, particularly owing to the underrepresentation of silent α-thalassaemia carriers in the dataset, such as individuals with genotypes αα/-α3.7 or αα/ααα, which often remain undetected through the population screening programme. Importantly, the -α3.7 deletion is the most common α-thalassaemia allele in Cyprus with a relative frequency of 72.8%, as reported in an earlier study that utilised a random dataset^7^. Nevertheless, the present study, through the compilation of a large dataset, reports the widest spectrum of α-globin gene mutations observed in the population since 1995, with several α-globin gene mutations reported for the first time in Cyprus. More specifically, three α-thalassaemia mutations, namely --SEA, -α4.2 and CD 108 (-C), and two α-chain structural variants, namely Hb Icaria and Hb Stanleyville-II, are reported for the first time through this study, whilst three cases with α^0^ deletions in the erythroid-specific DNAse I hypersensitive site MCS-R2 (HS40) were detected and are currently under investigation to determine the precise breakpoints. Moreover, four cases with unknown duplications of the α-locus and three cases with unknown deletions involving both genes, α2 and α1, were detected and are currently under investigation.

In addition, the geographic distribution of α-globin gene mutations reveals statistically significant differences between districts. Notably, the --MED I deletion is more prevalent in Larnaca/Famagusta, where the -α3.7 allele is less prevalent. Moreover, the severe IVS I-1 (-5 bp) allele is more prevalent in Larnaca/Famagusta and Paphos, while a higher prevalence of the -α20.5 deletion is observed in Nicosia.

### Carriers of β-globin gene mutations

Population screening in Cyprus was established mainly to prevent β-thalassaemia, which is usually a more severe disorder than the Hb H disease. For this reason, prenatal diagnosis has been offered for couples at risk for an affected β-thalassaemia birth. The list of all couples participating in prenatal testing is a random representation, with regards to genotype, of the β-thalassaemia carrier population in Cyprus, because it comprises unrelated individuals without any bias for mild or severe mutations. Thus, from the 4700 carriers of β-globin gene mutations genotyped at the CING during 1995–2015, we have selected all couples participating in prenatal testing for the calculation of the carrier frequencies, resulting in 2335 individuals, and the results are shown in Table 3. The most common β-thalassaemia mutation in the population is IVS I-110 (G>A), with a frequency of 79.01%, followed by mutations IVS I-6 (T>C), IVS I-1 (G>A) and IVS II-745 (C>G) with frequencies of 6.34%, 6.00% and 4.11%, respectively. These frequencies are in agreement with values reported in an earlier study^19^, but the present study provides both a more precise estimate through its much larger sample size and a critical update to numbers that date back 23 years.

Most geographical differences observed in β-globin gene mutation frequencies are small and not statistically significant, as demonstrated by the 95% CI shown in Table 3. Specifically, the frequency of the IVS I-110 (G>A) mutation is significantly higher in Paphos, albeit with a smaller sample size. In addition, small differences are observed in Limassol, with slightly higher frequencies for alleles IVS I-1 (G>A) and IVS II-745 (C>G), and Nicosia, with a slightly higher frequency for the IVS I-6 (T>C) allele.

Notably, several mutations are reported here for the first time in the Cypriot population. More specifically, five β-thalassaemia mutations, namely -87 (C>G), CD 44 (-C), IVS II-1 (G>A), -101 (C>T) and (δβ)^0^ Sicilian, and the β-chain structural variant Hb City of Hope have not been observed in earlier studies^19^,^28^, whereas the mutation IVS II-848 (C>A) has been reported only in the Turkish Cypriot population in the past^29^. Apart from the mutations listed in Table 3, other mutations were observed in individuals genotyped at the CING, but not included in the final dataset that was based on individuals participating in prenatal diagnosis. These mutations are rare in the population, with frequencies of 0.5% or lower, and include CD 8 (-AA), Hb Beirut, Hb Serres, Hb Limassol, Hb Nicosia, Hb O-Arab, Hb G-Accra that have been observed in the population in the past^19^,^22^, but also mutations that are reported in the Cypriot population for the first time, namely Hb C, Hb E, -30 (T>A), CD 36/37 (–T) and -92 (C>T).

### Carriers of δ-globin gene mutations

504 individuals carrying at least one δ-globin gene mutation were genotyped at the CING from 1995 to 2015. After removing related individuals, a dataset of 428 carriers was available to calculate the carrier frequencies shown in Table 4. The most common δ-globin gene mutation in Cyprus was CD 27 (GCC>TCC), resulting in Hb A_2_-Yialousa, with a frequency of 48.12%, followed by CD 4 (ACT>ATT), Hb A_2_-Yokoshima, Hb A_2_-Pelendri and IVS II-897 (A>G) with frequencies of 19.87%, 11.26%, 6.84% and 5.96%, respectively. Notably, three δ-globin chain variants were observed in the Cypriot population for the first time, namely Hb A_2_-NYU, Hb A_2_′ and Hb A_2_-Etolia.

Differences in the geographic distribution of δ-globin gene mutations are observed across different districts. Hb A_2_-Yialousa is particularly prevalent in the Larnaca/Famagusta district, while the CD 4 (ACT>ATT) allele is less prevalent. In Limassol, higher frequencies of Hb A_2_-Yokoshima and Hb A_2_-Pelendri are observed compared to Nicosia and Larnaca/Famagusta, respectively.

### β-thalassaemia patients

All known live 592 β-thalassaemia patients in Cyprus were genotyped at the CING and, with a population of 659115 Greek Cypriots (accounting for 98.8% of the total population), the prevalence of β-thalassaemia patients is estimated to be around 0.9 cases per 1000 people. Despite the application of a successful prevention programme, the prevalence is only slightly lower than earlier estimates of 1 in 1000^4^, which can be mainly attributed to the better survival of β-thalassaemia patients^30^. In addition, it was recently reported that the prevalence of β-thalassaemia carriers has been decreasing over the past 25 years, from an estimated 15–18% to around 12% of the population^5^, so that the present population of β-thalassaemia patients (average age of 41 years) is representative of a previously higher carrier rate.

Table 5 shows the relative allele and genotype frequencies in β-thalassaemia patients, including frequencies for each individual district. Notably, five mutations account for more than 95% of all β-thalassaemia alleles in the patient population. As expected, the most common β-globin gene mutation is IVS I-110 (G>A), with a percentage of 72.72%, followed by alleles IVS I-6 (T>C) and IVS I-1 (G>A), with frequencies of 12.42 and 5.15%, respectively. Consequently, homozygosity for IVS I-110 (G>A) is the most common genotype in β-thalassaemia patients with a frequency of 52.7%, while 18.92% of the patients are compound heterozygous for IVS I-110 (G>A) and IVS I-6 (T>C), with the latter allele usually associated with a milder phenotype than IVS I-110 G>A^16^. Notably, the frequency of IVS I-110 (G>A) is lower in patients than in carriers, while the frequency of IVS I-6 (T>C) is much higher, possibly due to a better survival rate in patients carrying the milder IVS I-6 (T>C) mutation.

### Hb H disease patients

For the first time, we have identified all 595 known Hb H disease patients in Cyprus and, thus, the prevalence of Hb H disease in the population is around 0.9 cases per 1000 persons. Hb H disease is caused by a combination of α^0^- and α^+^-thalassaemia alleles, and Table 6 shows the frequencies of α^0^ and α^+^ mutations and the observed genotype frequencies in Hb H disease patients in Cyprus and in individual districts. Two common alleles, namely -α3.7 and IVS I-1 (-5 bp), account for more than 95% of all α^+^ mutations and, similarly, deletions --MED I and -α20.5 account for more than 98% of all α^0^ mutations. Notably, the --MED I deletion and the IVS I-1 (-5 bp) allele are particularly prevalent in the east part of the island, namely in the Larnaca/Famagusta districts, while the -α20.5 deletion is more common in Nicosia than in other districts, mirroring similar observations in the α-thalassaemia carriers dataset (Table 2). Compound heterozygosity of α^0^ mutations with the -α3.7 deletion is the most common genotype, with a frequency of 82.35%. Importantly, compound heterozygosity of --MED I or -α20.5 with the IVS I-1 (-5 bp) α^+^-thalassaemia mutation, a nondeletional form of Hb H disease that is often assosiated with a more severe phenotype, is observed in 13.11% of the patients.

### Geographic distribution of haemoglobinopathies

**Figure 1:**
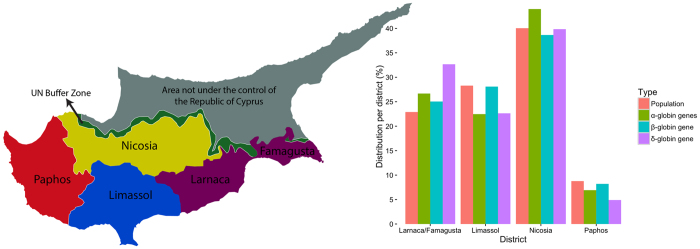
The distribution of carriers of α-, β- and δ-globin gene mutations per district, compared to the distribution of population in each district in Cyprus. The map of Cyprus was generated using the free web-tool Pixel Map Generator (http://pixelmap.amcharts.com) and was subsequently modified using Pixelmator v3.4.1 (http://www.pixelmator.com/mac) for OSX. The chart on the right was created using R (version 3.2.4).

Important differences are observed in the molecular spectrum of haemoglobinopathies across different districts, as discussed in previous sections, even though Cyprus is a small island with a total area of 9251 km^2^. In addition to the district-specific molecular spectra, differences are observed in the prevalence of haemoglobinopathies in different districts, as illustrated in Fig. 1. The figure shows the fraction of the population living in different districts, according to the latest population census, compared to the fraction of carriers of α-, β- and δ-globin gene mutations in the same districts, as reflected through the datasets used in this study, thus indicating under- and over-representation of different types of haemoglobinopathies relative to the share of the total population. Prevalence of haemoglobinopathies is generally higher than the island average in the Larnaca/Famagusta district, particularly for carriers of α- and δ-globin gene mutations, while an overrepresentation of carriers for α-globin gene mutations, only, is observed in Nicosia. In contrast, Limassol, the second-most populous district in Cyprus, has a lower prevalence of carriers of α- and δ-globin gene mutations and the same is observed in Paphos, the least populated district. As demonstrated in an earlier study for structural Hb variants^22^, a more detailed analysis of the geographic distribution of haemoglobinopathies would provide a valuable insight into the prevalence of thalassaemia mutations in Cyprus.

## Conclusions

This study represents the most significant update on the status of haemoglobinopathies in Cyprus for over two decades and reports the analysis of molecular data collected during 1995–2015 at the Molecular Genetics Thalassaemia department at the CING, the reference laboratory for genetic analysis in Cyprus. Notably, the total sample size of around 15000 individuals (patients and carriers) used in this study is much larger than the sample sizes used in previous studies^7^,^19^,^21^,^26^, making the current up-to-date report the most reliable investigation on the status and distribution of haemoglobinopathies in Cyprus. The estimated β-thalassaemia carrier rate is around 12–15%^4^,^5^ of the population, i.e. 80–100 thousands, and the α-thalassaemia carrier rate was estimated around 20%^7^, i.e. around 130 thousands. Thus, initial datasets of 9287 and 4700 carriers of α- and β-globin gene mutations, respectively, utilised in this study represent a significant fraction of the carrier population. Furthermore, this study demonstrates important differences in the prevalence and distribution of haemoglobinopathy alleles across different districts in Cyprus, with differences observed between the east and west part of the island. The analysis of the molecular spectrum and distribution of haemoglobinopathies provides valuable information to the population screening programme and facilitates effective prenatal diagnosis.

A limitation of this study is the lack of a random sample for the calculation of α-thalassaemia carrier frequencies, because a number of silent carriers are not detected by the population screening programme and, thus, are not referred to the CING for genetic analysis. Hence and because we analysed data collected through the routine molecular analysis at the CING since 1995, we could not compile a random dataset for the calculation of relative allele frequencies. The same limitation, however, is true for other studies of α-thalassaemia allele frequencies, even where this is not explicitly stated, mainly due to challenges involved in diagnosis^11^,^12^. A separate investigation should be performed on a random dataset to precisely determine the α-thalassaemia allele frequencies.

In addition and for the first time, we have identified and genotyped all thalassaemia patients in Cyprus, specifically β-thalassaemia and Hb H disease patients. To our knowledge, this is the first comprehensive analysis of the national β-thalassaemia population and Hb H disease population in any country. Hence, this study represents the first major step towards the challenging task to analyse and correlate genotype and phenotype for thalassaemia patients in Cyprus, which will be our future direction.

## Additional Information

**How to cite this article**: Kountouris, P. *et al*. The molecular spectrum and distribution of haemoglobinopathies in Cyprus: a 20-year retrospective study. *Sci. Rep.* **6**, 26371; doi: 10.1038/srep26371 (2016).

## Figures and Tables

**Table 1:** Description of datasets.

**Dataset**	**n**	**Per district (n)**^c^				**Sex (%)**		**Age (in years)**^d^		
		***Larnaca/ Famagusta***	***Limassol***	***Nicosia***	***Paphos***	***Male***	***Female***	***Mean***	***SD***	***Median***
*Patients*	1184									
β-thalassaemia	592	144	153	251	44	50.17	49.83	41.37	11.96	41
Hb H disease	595	209	98	256	13	49.16	50.84	36.87	19.71	34
*Carriers*	13824^a^									
α-thalassaemia	8412^b^ (out of 9287^a^)	2065	1739	3401	534	44.83	55.17	36.74	12.31	35
β-thalassaemia	2335^b^ (out of 4700^a^)	468	525	722	153	49.61	50.39	41.70	8.30	42
δ-thalassaemia	428^b^ (out of 504^a^)	127	88	155	19	47.90	52.10	38.89	12.54	35

^a^Including related individuals.

^b^After filtering related individuals with the same genotype.

^c^Number of individuals per district, after filtering those in unidentified locations.

^d^Age on 10 Dec 2015.

**Table 2:** Allele frequencies in 8412 carriers of α-globin gene mutations in Cyprus and in individual districts.

**Common name**	**HGVS name**	**IthaID**^1^	**Cyprus-wide**	**Per district (%) (95% CI)**			
			***n***	*Larnaca/Famagusta (n = 2415)*	***Limassol***(***n***** = *****2184***)	***Nicosia***(***n***** = *****4145***)	***Paphos***(***n***** = *****683***)
-α3.7 (type I)	NG_000006.1:g.34164_37967del3804	300	6363	50.60 (48.58, 52.61)	69.28 (67.29, 71.20)	64.32 (62.84, 65.77)	67.35 (63.67, 70.83)
--MED I	NG_000006.1:g.24664_41064del16401	312	1468	23.44 (21.77, 25.19)	9.98 (8.77, 11.33)	12.3 (11.33, 13.35)	8.78 (6.82, 11.22)
IVS I-1 (-5 bp)	HBA2:c.95 + 2_95 + 6delTGAGG	359	1458	17.02 (15.55, 18.59)	12.59 (11.24, 14.07)	13.29 (12.28, 14.37)	17.57 (14.83, 20.68)
-α20.5	NG_000006.1:g.15164_37864del22701	314	382	2.98 (2.36, 3.76)	2.61 (2.00, 3.39)	4.95 (4.32, 5.66)	1.76 (0.95, 3.14)
Poly(A) AATAAA>AATGAA	HBA2:c.*92A>G	425	212	2.15 (1.63, 2.84)	2.79 (2.16, 3.60)	1.93 (1.54, 2.41)	0.44 (0.11, 1.39)
Triplicated α (anti-3.7)	NA	2561	102	0.99 (0.65, 1.50)	0.6 (0.33, 1.04)	1.09 (0.80, 1.46)	1.02 (0.45, 2.20)
Hb Agrinio	HBA2:c.89T>C	356	86	1.20 (0.82, 1.74)	0.92 (0.57, 1.44)	0.58 (0.38, 0.87)	1.46 (0.75, 2.77)
Poly(A) AATAAA>AATAAG	HBA2:c.*94A>G	424	50	0.75 (0.46, 1.20)	0.32 (0.14, 0.69)	0.46 (0.28, 0.73)	0.73 (0.27, 1.80)
Hb Setif	HBA2:c.283G>T	673	39	0.17 (0.05, 0.45)	0.37 (0.17, 0.75)	0.51 (0.32, 0.79)	0.73 (0.27, 1.80)
CD 108 (-C)	HBA1:c.327delC	398	14	0.25 (0.10, 0.57)	0.09 (0.02, 0.37)	0.10 (0.03, 0.27)	0.15 (0.01, 0.95)
Hb Icaria	HBA2:c.427T>A	419	11	0.04 (0.00, 0.27)	0.05 (0.00, 0.30)	0.22 (0.11, 0.43)	–
--MED II	NG_000006.1:g.10864_40864del30001	313	8	0.25 (0.10, 0.57)	0.05 (0.00, 0.30)	0.02 (0.00, 0.16)	–
-α4.2	NA	301	5	0.08 (0.01, 0.33)	0.09 (0.02, 0.37)	0.02 (0.00, 0.16)	–
Hb Fontainebleau	HBA2:c.64G>C	481	4	–	0.14 (0.04, 0.44)	0.02 (0.00, 0.16)	–
Unknown duplications	NA	NA	4	–	0.09 (0.02, 0.37)	0.05 (0.01, 0.19)	–
HS40 deletions	NA	NA	3	0.04 (0.00, 0.27)	–	0.05 (0.00, 0.16)	–
Unknown deletions involving α_2_ and α_1_	NA	NA	3	0.04 (0.00, 0.27)	0.05 (0.00, 0.30)	0.02 (0.00, 0.16)	–
--SEA	NG_000006.1:g.26264_45564del19301	309	2	–	–	0.05 (0.00, 0.16)	–
Hb Stanleyville-II	HBA2:c.[237C>A; 237C>G]	626	1	–	–	0.02 (0.00, 0.16)	–

n: number of alleles; NA: Not available.

**Table 3:** Allele frequencies in 2335 carriers of β-globin gene mutations in in Cyprus and in individual districts.

**Common name**	**HGVS name**	**IthaID**^1^	**Cyprus-wide**		**Per district (%) (95% CI)**			
			***n***	*Frequency (%) (95% CI)*	*Larnaca/Famagusta (n = 468)*	***Limassol***(***n *****=***** 525***)	***Nicosia***(***n *****=***** 722***)	***Paphos***(***n *****=***** 153***)
IVS I-110 (G>A)	HBB:c.93-21G>A	113	1845	79.01 (77.29, 80.64)	79.49 (75.48, 83.00)	74.48 (70.48, 78.11)	77.42 (74.16, 80.39)	92.16 (86.39, 95.70)
IVS I-6 (T>C)	HBB:c.92 + 6T>C	111	148	6.34 (5.40, 7.42)	5.98 (4.08, 8.63)	6.48 (4.59, 9.02)	7.34 (5.60, 9.55)	3.27 (1.21, 7.86)
IVS I-1 (G>A)	HBB:c.92 + 1G>A	101	140	6.00 (5.08, 7.06)	6.84 (4.80, 9.62)	8.00 (5.89, 10.74)	5.40 (3.92, 7.38)	1.96 (0.51, 6.07)
IVS II-745 (C>G)	HBB:c.316-106C>G	214	96	4.11 (3.36, 5.02)	4.70 (3.04, 7.14)	5.33 (3.64, 7.71)	4.02 (2.75, 5.79)	1.96 (0.01, 0.89)
CD 39 (CAG>TAG)	HBB:c.118C>T	142	42	1.80 (1.32, 2.45)	0.21 (0.01, 1.38)	1.90 (0.97, 3.59)	3.05 (1.97, 4.65)	–
Hb S	HBB:c.20A>T	824	22	0.94 (0.61, 1.45)	0.85 (0.27, 2.32)	1.14 (0.47, 2.60)	0.97 (0.43, 2.08)	0.65 (0.03, 4.14)
-87 C>G	HBB:c.-137C>G	10	12	0.51 (0.28, 0.92)	0.43 (0.07, 1.71)	0.76 (0.24, 2.08)	0.42 (0.11, 1.32)	–
CD 44 (-C)	HBB:c.135delC	151	10	0.43 (0.22, 0.81)	–	1.14 (0.47, 2.60)	0.55 (0.18, 1.51)	–
Hb Lepore Boston-Washington	NG_000007.3:g.63632_71046del7414	1399	4	0.17 (0.05, 0.47)	0.21 (0.01, 1.38)	0.19 (0.01, 1.23)	0.14 (0.01, 0.89)	–
IVS II-848 (C>A)	HBB:c.316-3C>A	221	4	0.17 (0.05, 0.47)	0.85 (0.01, 1.38)	–	–	–
IVS II-1 (G>A)	HBB:c.315 + 1G>A	200	3	0.13 (0.03, 0.41)	0.21 (0.01, 1.38)	–	0.14 (0.01, 0.89)	–
Sicilian (δβ)^0^	NG_000007.3:g.64336_77738del13403	1507	3	0.13 (0.03, 0.41)	–	0.38 (0.07, 1.52)	–	–
CAP + 33 (C>G)	HBB:c.-18C>G	39	2	0.09 (0.01, 0.34)	–	0.19 (0.01, 1.23)	0.14 (0.01, 0.89)	–
-101 (C>T)	HBB:c.-151C>T	3	1	0.04 (0.002, 0.28)	–	–	0.14 (0.01, 0.89)	–
Hb City of Hope	HBB:c.208G>A	1030	1	0.04 (0.002, 0.28)	0.21 (0.01, 1.38)	–	–	–
Hb D-Punjab	HBB:c.364G>C	1217	1	0.04 (0.002, 0.28)	–	–	0.14 (0.01, 0.89)	–
Hb Knossos	HBB:c.82G>T	91	1	0.04 (0.002, 0.28)	–	–	0.14 (0.01, 0.89)	–

n: number of alleles.

**Table 4:** Allele frequencies in 428 carriers of δ-globin gene mutations in Cyprus and in individual districts.

**Common name**	**HGVS name**	**IthaID**^1^	**Cyprus-wide**		**Per district (%) (95% CI)**			
			***n***	*Frequency (%) (95% CI)*	*Larnaca/Famagusta (n = 138)*	***Limassol***(***n *****=***** 94***)	***Nicosia***(***n *****=***** 160***)	***Paphos***(***n *****=***** 19***)
Hb A_2_-Yialousa	HBD:c.82G>T	1332	218	48.12 (43.45, 52.83)	59.42 (50.72, 67.59)	24.47 (16.44, 34.62)	49.38 (41.43, 57.35)	36.84 (17.23, 61.37)
CD 4 (ACT>ATT)	HBD:c.14C>T	1331	80	19.87 (16.35, 23.91)	9.42 (5.32, 15.88)	28.72 (20.09, 39.12)	22.50 (16.44, 29.91)	36.84 (17.23, 61.37)
Hb A_2_-Yokoshima	HBD:c.77G>A	1360	51	11.26 (8.57, 14.62)	13.77 (8.70, 20.92)	20.21 (12.90, 30.01)	6.88 (3.65, 12.27)	5.26 (0.28, 28.11)
Hb A_2_-Pelendri	HBD:c.425T>C	1392	31	6.84 (4.77, 9.68)	3.62 (1.34, 8.68)	15.96 (9.50, 25.27)	5.00 (2.34, 9.95)	–
IVS II-897 (A>G)	HBD:c.316-2A>G	2473	27	5.96 (4.04, 8.66)	7.25 (3.72, 13.27)	7.45 (3.30, 15.25)	3.75 (1.53, 8.35)	5.26 (0.28, 28.11)
Hb A_2_-Famagusta	HBD:c.60C>A	2292	8	1.77 (0.82, 3.59)	1.45 (0.25, 5.67)	–	1.88 (0.49, 5.81)	15.79 (4.17, 40.49)
Hb A_2_-Troodos	HBD:c.349C>T	1384	5	1.10 (0.41, 2.71)	1.45 (0.25, 5.67)	1.06 (0.06, 6.62)	1.25 (0.22, 4.91)	–
IVS I-2 (T>C)	HBD:c.92 + 2T>C	1334	5	1.10 (0.41, 2.71)	0.72 (0.04, 4.57)	1.06 (0.06, 6.62)	1.25 (0.22, 4.91)	–
-55 (T>C)	HBD:c.-105T>C	1326	4	0.88 (0.28, 2.40)	1.45 (0.25, 5.67)	–	1.25 (0.22, 4.91)	–
CD 59 (-A)	HBD:c.178delA	1337	4	0.88 (0.28, 2.40)	–	–	2.50 (0.80, 6.68)	–
Hb A_2_-Wrens	HBD:c.295G>A	1339	4	0.88 (0.28, 2.40)	–	–	2.50 (0.80, 6.68)	–
-30 (T>C)	HBD:c.-80T>C	1329	2	0.44 (0.08, 1.76)	–	–	1.25 (0.22, 4.91)	–
Hb A_2_-NYU	HBD:c.39T>A	1354	2	0.44 (0.08, 1.76)	0.72 (0.04, 4.57)	–	0.62 (0.03, 3.96)	–
Hb A_2_′ (or Hb B2)	HBD:c.49G>C	1356	1	0.22 (0.01, 1.42)	–	1.06 (0.06, 6.62)	–	–
Hb A_2_-Etolia	HBD:c.257T>C	1376	1	0.22 (0.01, 1.42)	0.72 (0.04, 4.57)	–	–	–

n: number of alleles.

**Table 5:** Allele frequencies and genotypes in 592 β-thalassaemia patients in Cyprus and in individual districts.

**Mutation/Genotype**	**Cyprus-wide**		**Per district (%)**			
	***n***	***Frequency***(***%***)	***Larnaca/Famagusta***	***Limassol***	***Nicosia***	***Paphos***
*β-globin mutations*						
IVS I-110 (G>A)	861	72.72	75.69	74.51	68.33	81.82
IVS I-6 (T>C)	147	12.42	12.85	10.13	14.14	9.09
IVS I-1 (G>A)	61	5.15	5.56	5.23	5.18	3.41
IVS II-745 (C>G)	41	3.46	3.47	2.29	4.18	3.41
CD 39 (CAG>TAG)	28	2.36	0.35	2.94	3.39	1.14
Hb S	20	1.69	0.35	1.96	2.39	1.14
-87 (C>G)	12	1.01	1.39	1.63	0.60	–
CAP + 33 (C>G)	6	0.51	–	–	1.20	–
IVS II-1 (G>A)	4	0.34	–	0.65	0.40	–
CD 44 (-C)	2	0.17	–	0.65	–	–
-86 (C>G)	1	0.08	–	–	0.20	–
CD 5 (-CT)	1	0.08	0.35	–	–	–
*β-globin genotypes*						
IVS I-110 (G>A) / IVS I-110 (G>A)	312	52.70	59.03	52.29	47.41	63.64
IVS I-110 (G>A) / IVS I-6 (T>C)	112	18.92	19.44	16.99	19.92	18.18
IVS I-110 (G>A) / IVS I-1 (G>A)	47	7.94	6.25	9.15	8.37	6.82
IVS I-110 (G>A) / IVS II-745 (C>G)	27	4.56	4.17	4.58	4.38	6.82
IVS I-110 (G>A) / CD 39 (CAG>TAG)	21	3.55	–	5.88	4.38	2.27
IVS I-110 (G>A) / Hb S	13	2.20	0.69	3.27	2.39	2.27
IVS I-6 (T>C) / IVS I-6 (T>C)	12	2.03	2.08	1.31	2.79	–
IVS I-110 (G>A) / -87 (C>G)	9	1.52	2.08	3.27	0.40	–
IVS I-1 (G>A) / IVS II-745 (C>G)	6	1.01	2.08	–	1.20	–
CAP + 33 (C>G) / CD 39 (CAG>TAG)	4	0.68	–	–	1.59	–
IVS I-110 (G>A) / IVS II-1 (G>A)	4	0.68	–	1.31	0.80	–
IVS I-6 (T>C) / IVS II-745 (C>G)	4	0.68	0.69	–	1.20	–
IVS I-1 (G>A) / IVS I-6 (T>C)	3	0.51	0.69	–	0.80	–
Hb S/Hb S	2	0.34	–	–	0.80	–
IVS I-110 G>A / CAP + 33 (C>G)	2	0.34	–	–	0.80	–
IVS I-6 (T>C) / Hb S	2	0.34	–	0.65	0.40	–
-87 C>G / CD 39 (CAG>TAG)	1	0.17	–	–	0.40	–
CD 44 -C / IVS I-1 G>A	1	0.17	–	0.65	–	–
IVS I-1 G>A / -87 C>G	1	0.17	0.69	–	–	–
IVS I-1 G>A / CD 44 –C	1	0.17	–	0.65	–	–
IVS I-1 G>A / IVS I-1 G>A	1	0.17	0.69	–	–	–
IVS I-110 G>A / -86 C>G	1	0.17	–	–	0.40	–
IVS I-110 G>A / CD 5 -CT	1	0.17	0.69	–	–	–
IVS I-6 (T>C) / -87 C>G	1	0.17	–	–	0.40	–
IVS I-6 (T>C) / CD 39 (CAG>TAG)	1	0.17	0.69	–	–	–
IVS II-745 C>G / CD 39 (CAG>TAG)	1	0.17	–	–	0.40	–
IVS II-745 C>G / Hb S	1	0.17	–	–	0.40	–
IVS II-745 C>G / IVS II-745 C>G	1	0.17	–	–	0.40	–

**Table 6:** Frequencies of α-globin gene mutations and genotypes in 595 Hb H disease patients in Cyprus and in individual districts.

**Mutation/Genotype**	**Cyprus-wide**		**Per District (%)**^**a**^			
	***n***	***Frequency***(***%***)	***Larnaca/Famagusta***	***Limassol***	***Nicosia***	***Paphos***
*α*^+^ *mutations*						
IVS I-1 (-5 bp)	78	13.11	17.22	7.14	12.5	7.69
Poly(A) AATAAA>AATGAA	17	2.86	0.96	8.16	1.95	–
CD 108 (-C)	5	0.84	0.48	1.02	0.78	7.69
Hb Icaria	3	0.50	–	2.04	0.39	–
-α4.2	1	0.17	–	–	0.39	–
Hb Agrinio	1	0.17	–	–	0.39	–
*α*^*0*^ *mutations*						
--MED I	483	81.18	89.47	79.59	73.83	92.31
-α20.5	105	17.65	10.53	16.33	25.00	7.69
HS40 deletion	3	0.50	–	2.04	0.39	–
--MED II	2	0.34	–	2.04	–	–
--SEA	2	0.34	–	–	0.78	–
*α-globin genotypes*						
-α3.7 / --MED I	396	66.55	73.21	64.29	61.33	76.92
-α3.7 (type I) / -α20.5	88	14.79	8.13	14.29	21.09	7.69
IVS I-1 (-5 bp) / --MED I	64	10.76	14.83	6.12	9.38	7.69
IVS I-1 (-5 bp) / -α20.5	14	2.35	2.39	1.02	3.12	–
Poly(A) AATAAA>AATGAA / --MED I	13	2.18	0.96	6.12	1.17	–
CD 108 (-C) / --MED I	5	0.84	0.48	1.02	0.78	7.69
-α3.7 (type I) / HS40 deletion	3	0.50	–	2.04	0.39	–
Poly(A) AATAAA>AATGAA / -α20.5	3	0.50	–	1.02	0.78	–
Hb Icaria / --MED I	3	0.50	–	2.04	0.39	–
-α3.7 (type I) / --SEA	2	0.34	–	–	0.78	–
-α3.7 (type I) / --MED II	1	0.17	–	1.02	–	–
-α4.2 / --MED I	1	0.17	–	–	0.39	–
Hb Agrinio / --MED I	1	0.17	–	–	0.39	–
Poly(A) AATAAA>AATGAA / --MED II	1	0.17	–	1.02	–	–

## References

[b1] Kountouris P. . IthaGenes: An Interactive Database for Haemoglobin Variations and Epidemiology. PLoS ONE 9, e103020 (2014). 25058394 10.1371/journal.pone.0103020 PMC4109966

[b2] Modell B. & Darlison M. Global epidemiology of haemoglobin disorders and derived service indicators. Bull. World Health Organ. 86, 480–487 (2008). 18568278 10.2471/BLT.06.036673 PMC2647473

[b3] Cappellini M. D. . Guidelines for the clinical management of thalassaemia. 1–202 (Thalassaemia International Federation, 2008). 24308075

[b4] Angastiniotis M. A., Kyriakidou S. & Hadjiminas M. How thalassaemia was controlled in Cyprus. World health forum 7 **(3)**, 291–297 (1986).

[b5] Kyrri A. R. . The changing epidemiology of β-thalassemia in the Greek-Cypriot population. Hemoglobin 37, 435–443 (2013). 24006929 10.3109/03630269.2013.801851

[b6] Angastiniotis M., Kyriakidou S. & Hadjiminas M. The Cyprus Thalassemia Control Program. Birth Defects Orig. Artic. Ser. 23, 417–432 (1988). 3390571

[b7] Kyriacou K. . Hb Bart’s levels in cord blood and alpha-thalassemia mutations in Cyprus. Hemoglobin 24, 171–180 (2000). 10975437 10.3109/03630260008997525

[b8] Ashiotis T., Zachariadis Z., Sofroniadou K., Loukopoulos D. & Stamatoyannopoulos G. Thalassaemia in Cyprus. British Medical Journal 2, 38–42 (1973). 4695698 10.1136/bmj.2.5857.38 PMC1588975

[b9] Hadjiminas M., Zachariadis Z. & Stamatoyannopoulos G. Alpha-thalassaemia in Cyprus. J. Med. Genet. 16, 363–365 (1979). 513081 10.1136/jmg.16.5.363 PMC1012610

[b10] Angastiniotis M. A. & Hadjiminas M. G. Prevention of thalassaemia in Cyprus. The Lancet 1, 369–371 (1981). 10.1016/s0140-6736(81)91682-2 6109998

[b11] Galanello R. & Cao A. Alpha-thalassemia. Genet Med 13, 83–88 (2011). 21381239 10.1097/GIM.0b013e3181fcb468

[b12] Piel F. B. & Weatherall D. J. The α-Thalassemias. N Engl J Med 371, 1908–1916 (2014). 25390741 10.1056/NEJMra1404415

[b13] Old J. . Prevention of Thalassaemias and Other Haemoglobinopathies. 1, (Thalassaemia International Federation, 2013).

[b14] Chui D. H. K., Fucharoen S. & Chan V. Hemoglobin H disease: not necessarily a benign disorder. Blood 101, 791–800 (2003). 12393486 10.1182/blood-2002-07-1975

[b15] Felekis X. . Hb Agrinio [alpha29(B10)Le– >uPro (alpha2)] in combination with –(MED I). Results in a severe form of Hb H disease. Hemoglobin 32, 237–246 (2008). 18473239 10.1080/03630260802004103

[b16] Galanello R. & Origa R. Beta-thalassemia. Orphanet J Rare Dis 5, 11 (2010). 20492708 10.1186/1750-1172-5-11 PMC2893117

[b17] Thein S. L. The molecular basis of β-thalassemia. Cold Spring Harbor Perspectives in Medicine 3, a011700 (2013). 23637309 10.1101/cshperspect.a011700 PMC3633182

[b18] Cao A. & Galanello R. Beta-thalassemia. Genet Med 12, 61–76 (2010). 20098328 10.1097/GIM.0b013e3181cd68ed

[b19] Baysal E. . The β-thalassaemia mutations in the population of Cyprus. Br. J. Haematol. 81, 607–609 (1992). 1390250 10.1111/j.1365-2141.1992.tb03000.x

[b20] Trifillis P. . Analysis of delta-globin gene mutations in Greek cypriots. Blood 82, 1647–1651 (1993). 8364213

[b21] Pavlou E. . δ-Thalassemia in Cyprus. Hemoglobin 30, 455–462 (2006). 16987800 10.1080/03630260600868006

[b22] Kyrri A. R. . Hemoglobin Variants in Cyprus. Hemoglobin 33, 81–94 (2009). 19373583 10.1080/03630260902813502

[b23] Lederer C. W. . Hb Famagusta–analysis of a novel δ-globin chain variant [HBD:c.60C >A] in four families with diverse globin genotypes. Annals of Hematology 93, 1625–1627 (2014). 24452365 10.1007/s00277-013-1996-6

[b24] Old J. M., Varawalla N. Y. & Weatherall D. J. Rapid detection and prenatal diagnosis of beta-thalassaemia: studies in Indian and Cypriot populations in the UK. The Lancet 336, 834–837 (1990). 10.1016/0140-6736(90)92338-i 1976877

[b25] Goossens M. . Triplicated alpha-globin loci in humans. Proceedings of the National Academy of Sciences 77, 518–521 (1980). 10.1073/pnas.77.1.518 PMC348303 6928643

[b26] Baysal E. . α-Thalassaemia in the population of Cyprus. Br. J. Haematol. 89, 496–499 (1995). 7734346 10.1111/j.1365-2141.1995.tb08354.x

[b27] Lederer C. W. . An electronic infrastructure for research and treatment of the thalassemias and other hemoglobinopathies: the Euro-mediterranean ITHANET project. Hemoglobin 33, 163–176 (2009). 19657830 10.1080/03630260903089177

[b28] Traeger-Synodinos J., Maragoudaki E., Vrettou C., Kanavakis E. & Kattamis C. Rare beta-thalassemia alleles in the Greek and Greek Cypriot populations. Hemoglobin 22, 89–94 (1998). 9494054 10.3109/03630269809071524

[b29] Tüzmen S., Basak A. N. & Baysal E. Rare β‐thalassemia mutation IVS‐II‐848 (C‐A) first reported in a Turkish cypriot family. Am. J. Hematol. 54, 338–339 (1997). 9092695

[b30] Telfer P. . Survival of medically treated thalassemia patients in Cyprus. Trends and risk factors over the period 1980–2004. Haematologica 91, 1187–1192 (2006). 16956817

